# Z-Plasty Made Simple

**DOI:** 10.1155/2010/982623

**Published:** 2011-06-26

**Authors:** Sumaira Z. Aasi

**Affiliations:** Section of Dermatologic Surgery and Cutaneous Oncology, Department of Dermatology, Yale University School of Medicine, New Haven, CT 06510, USA

## Abstract

A Z-plasty is a critical and reliable technique that is useful for scar revisions and correction of free margin distortion. A Z-plasty can help lengthen a contracted scar, change the direction of a scar so that it is better aligned with the relaxed skin tension lines, or interrupt and break a scar for better camouflage. This article will review the technique of a basic Z-plasty as well as provide case examples of its use in free margin distortion and scar revision.

## 1. Introduction

In order to achieve optimal aesthetic and functional results, there is no substitute for careful planning and meticulous surgical technique. Yet even in the most competent hands, surgical complications are an inevitable fact of life. In particular, areas of the face that border free margins, such as the eyes, nose, and lips, present a special challenge to the dermatologic surgeon. These areas offer little resistance to any tension created by surgical movement of nearby tissue and are not very forgiving. Any abnormality in the natural contours of these visually critical structures focuses the attention to the disruption or distortion itself. Often the distortion not only leads to an unacceptable cosmetic result but may also have functional consequences.

 A Z-plasty [1] is a critical and reliable technique for the dermatologic surgeon when performing revisions or correcting free margin distortion. There are three main objectives when performing a Z-plasty: to lengthen a contracted scar, to change the direction of a scar so that it is better aligned with the relaxed skin tension lines, or to interrupt and break a scar for better camouflage. When it comes to free margins, a Z-plasty is particularly useful because it does not require excising more tissue which is often at a premium near free margins. 

## 2. Design and Execution

**Figure 1:**
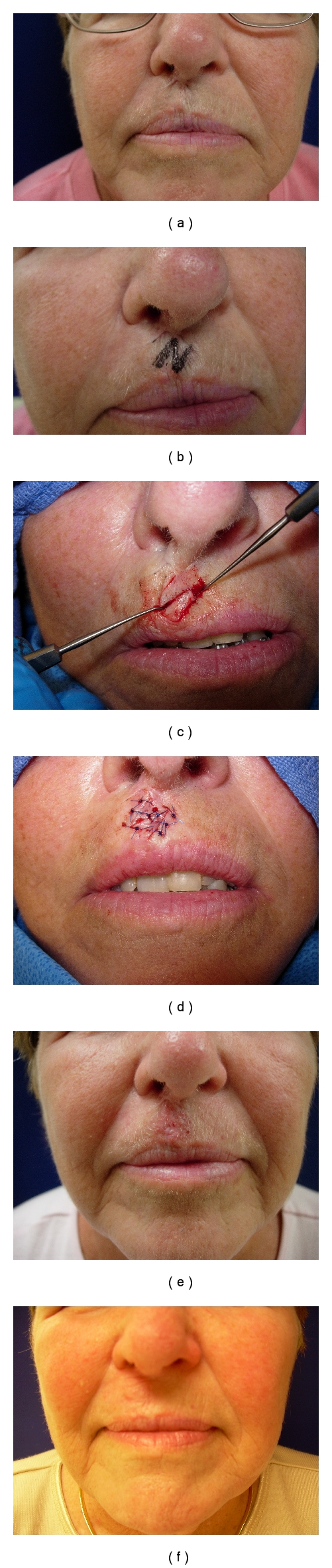
(a) Complication of eclabium secondary to herpetic infection of an advancement flap of the upper lip. (b) A 45-degree Z-plasty is planned and drawn. (c) The flaps are widely undermined and transposed. Note the use of skin hooks to minimize trauma to the tenuous tips. (d) Flaps sutured in place. Transposition of the triangular flaps brings about the following changes: the central limb is rotated (from a vertical to a horizontal direction), the distance between the original vertical scar (or limb) is increased, and the final scar is “broken” from a straight line to a nonlinear Z configuration. (e) Patient at suture removal. Note the immediate correction of the eclabium. (f) Results at several months. The scar lines have become more subtle.

As with all surgical reconstruction, it is essential to carefully plan, measure, and draw out the Z-plasty. Ideally this is done before any local anesthesia is injected to prevent tissue distortion. The fundamental unit of a Z-plasty is a triangular double transposition flap. However, as with most lifting flaps, there are also elements of rotation and advancement. In a classic Z-plasty three incisions of equal length create two equilateral triangular flaps (see Figures 1(b) and 1(c)). There is a central limb which is either the scar itself that needs to be redirected or an incision parallel to this scar. There are also two arms that originate from this central limb at various angles. Ideally, the angles may be measured using a protractor or they can be estimated free hand. That is, one can draw a 90-degree limb that comes off the central limb and then either bisect it to create a 45-degree angles or trisect it to create a 30- and, the more commonly used, 60-degree angles. Precise, confident incisions and delicate tissue handling are critical because the triangular flaps that are being excised and transposed are often rather small. 

Undermining as in any transposition flap is also a key so that the flap can be transposed without tension. When trying to free a contracture or redirect a scar, it is especially important to undermine widely below any fibrosis that constrains the scar. If one only undermines above this plane, the flaps will transpose but the scar tissue below the flaps will prevent release of the contracture and will not significantly redirect or lengthen the original scar. In addition, for the novice, it may be helpful to place an indelible dot of ink on one of the flap tips. It is easy to sew the flaps back into their original position and not actually transpose them once they have been undermined and freed (since suturing the flaps with or without transposing will create a z or mirror image of a z). After undermining, one triangular flap is transposed about its pivotal point in a clockwise direction and the other in a counterclockwise direction to create the Z-plasty. In most cases, one does not need a lot of buried interrupted sutures and must be fairly gentle with the tips of the Z-plasty to avoid vascular compromise of these small flaps [2].

## 3. Limitations and Considerations

**Figure 2:**
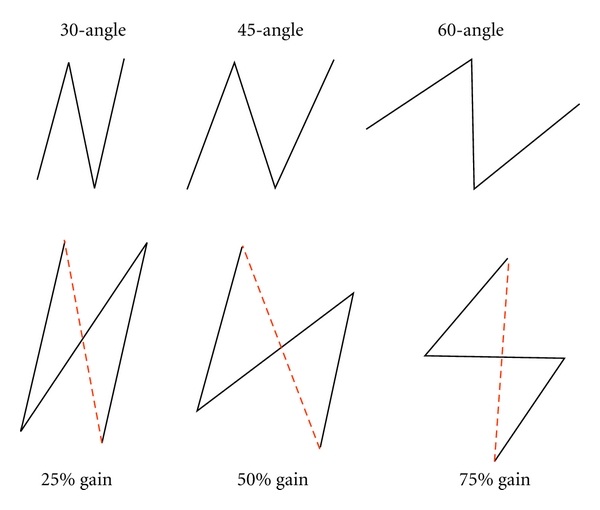
The degree of scar directional change and lengthening is affected by the angles of the flap. In the 30-degree Z-plasty, the direction of the change is approximately 45 degrees from the initial position of the central limb and there is a 25% gain in scar length. With a 45-degree Z-plasty, the resultant limb lies in a more oblique position, approximately 60 degrees from the initial position and there is a 50% gain in scar length. A 60-degree Z-plasty will yield a 75% increase in scar length and a 90-degree change in scar direction (the broken line represents the original scar).

The degree of scar directional change and lengthening is determined by the angles of the flap: the larger the angle, the greater the lengthening and the greater the change in direction of the original scar, the smaller the angle, the less the lengthening and change in the direction of the original scar (Figure 2). Although angles between 30 and 90 degrees are possible, the 60-degree Z-plasty is most common. A 60-degree Z-plasty will yield a 75% increase in scar length and a 90-degree change in scar direction. In the 45-degree Z-plasty, the resultant central limb lies in a more oblique position, approximately 60 degrees from the initial position. In the 30-degree Z-plasty, the direction of the change is even less, approximately 45 degrees from the initial position of the central limb. Z-plasties with angles less than 30 degrees will greatly increase the risk of tip necrosis. Z-plasties created by angles greater than 60 degrees will make it more difficult to transpose the two flaps without tension and also produce standing cutaneous deformities or “dog ears.” It is important to bear in mind that these mathematical predictions do not bear out precisely in practice when dealing with live tissue because of factors such as pliability and tensile forces that place constraints on skin. Thus, the gain in scar lengthening or directional change is almost always less than calculated. 

**Figure 3:**
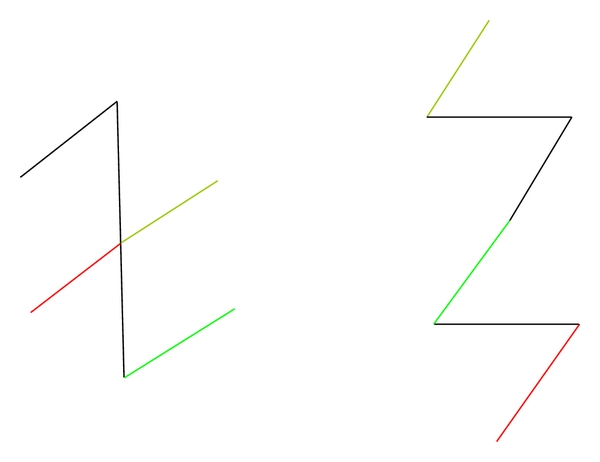
A multiple Z-plasty.

The limb lengths of the Z-plasty also influence the length gained: the longer the limbs, the greater the gain in scar length. Yet these limbs of the Z-plasty have some practical limitations in terms of their length. Although the broken line scar created by a Z-plasty allows for an overall less perceptible scar, this advantage diminishes with longer limbs as they will eventually create more obvious scar lines (and an unnatural appearance of a Z-shaped scar). Usually 6 to 10 millimeters is as long as any segment of the Z-plasty should be designed, particularly if the segment does not lie in a favorable direction or at the junction of two cosmetic units. In order to decrease the formation of a noticeable Z-shaped scar and overcome the practical limb lengths, a longer scar can be broken up by using multiple Z-plasties (Figure 3). Finally, mechanical dermabrasion or laser resurfacing can be performed afterwards to camouflage and make the Z-shaped scar more subtle. 

**Figure 4:**
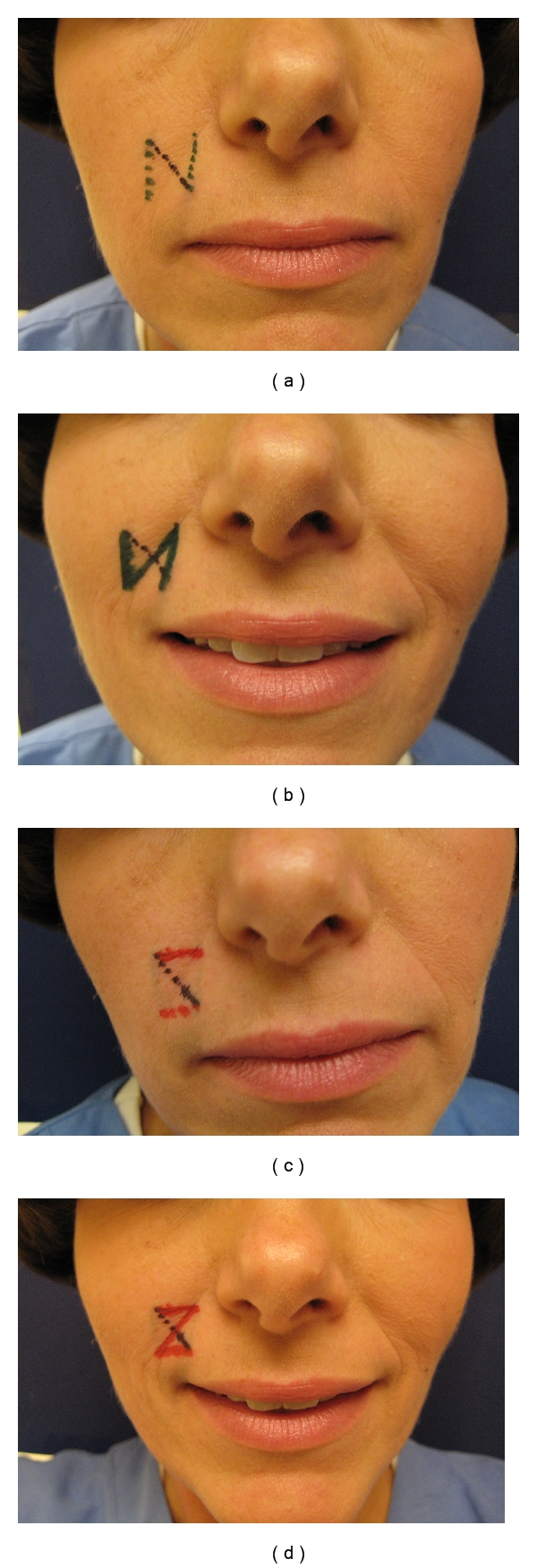
For a scar that crosses the melolabial fold (black line) in an undesirable perpendicular fashion, there are two ways that the Z-plasty could be performed to change the scar direction (represented by the green and red inks in Figures 4(a) and 4(c)). Although both options will change the scar direction to match the melolabial fold, only one of the designs (green ink Figure 4(b)) allows for the limbs of the Z-plasty to align with the RSTLs of the cheek and lip. Conversely, in Figure 4(d) even though the mirror image also realigns the central limb appropriately, the resultant lateral limbs cross the RSTL of the lip and cheek in an undesirable perpendicular fashion.

Finally one should also consider the relaxed skin tension lines of the face when planning the Z-plasty such that the amount of directional change (and hence final scar lines) can be most similar to the RSTLs. For each particular scar or central limb, there are always two choices for the alignment of the peripheral arms, these being mirror images of one another (Figure 4). Both options should be considered since both designs will change the scar direction; however, one of these options will usually be a better choice for scar camouflage due to placement of the lateral limbs such that they more correctly mimic the RSTL.

Like other transposition flaps, a Z-plasties can create trapdoor or pincushion effect. Wide undermining to create an even scar contraction plate may help decrease this risk. Post-operative intralesional steroids may also help diminish such an effect should it occur.

**Case Examples**

**Case 1**

**Figure 5:**
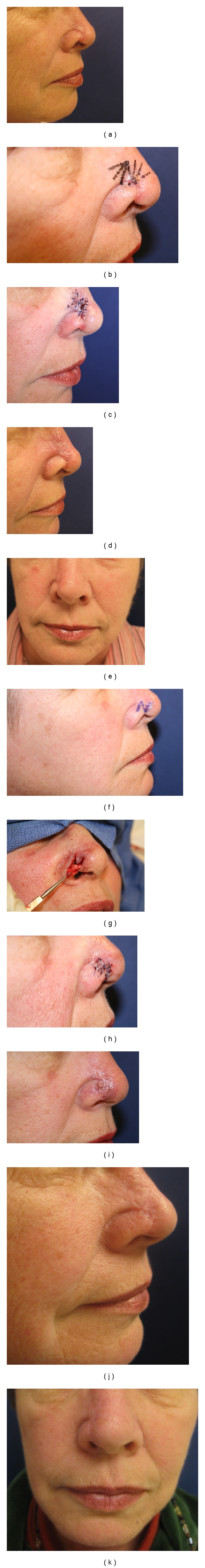
(a) Alar retraction secondary to multiple tumor extirpations resulting in an ecnasion. (b) A Z-plasty is planned. One relatively simple way to estimate the angles is to draw out the peripheral arms at 90 angles to the central limb and then divide this in 1/2 to get 45-degree angles or trisect it to obtain 30- or 60-degree angles as shown. (c) Z-plasty sutured in place. ((d) and (e)) Frontal and lateral views demonstrating that alar retraction has improved but not completely resolved (cf.  Figure 5(a)). (f) Another, more inferior Z-plasty is planned at a later date. (g) A strip of cartilage is used underneath the flap to provide structural support to the alar rim. (h) Z-plasty sutured in place. (i) Patient at suture removal. (j-k) Frontal and lateral views demonstrating resolution of the ecnasion.

Figure 5 represents an example of Case 1.

**Case 2**

**Figure 6:**
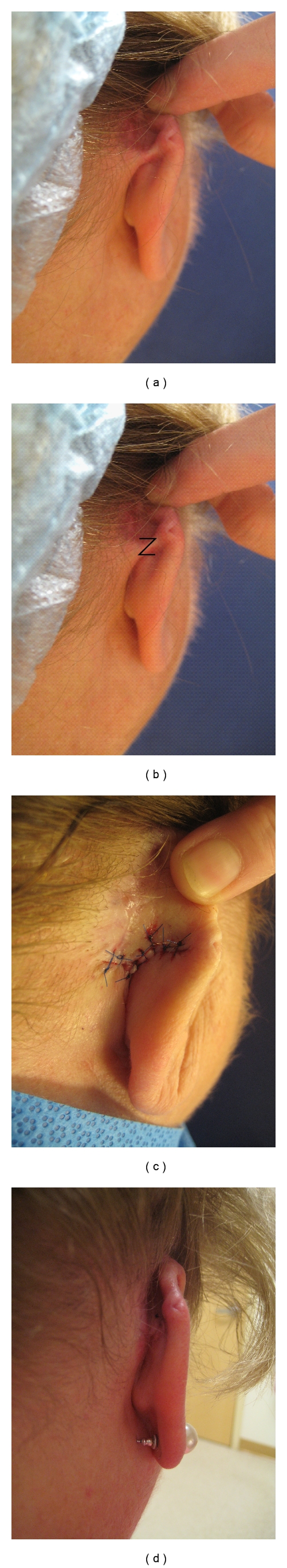
(a) Scar creating webbing in the retroauricular sulcus. (b) Z-plasty designed. (c) A Z-plasty is performed to release the contracture. (d) Patient at suture removal.

Figure 6 represents an example of Case 2.
